# Supplementary material for: Specific stereochemistry of OP-1074 disrupts estrogen receptor alpha helix 12 and confers pure antiestrogenic activity
Source: Nat Commun. 2018 Jun 18;9:2368. doi: 10.1038/s41467-018-04413-3 (PMC6006285; doi:10.1038/s41467-018-04413-3)
Supplement: Supplementary file 1 — Supplementary Information [file 41467_2018_4413_MOESM1_ESM.pdf]

Supplementary Information

Specific stereochemistry of OP-1074 disrupts estrogen receptor alpha helix 12 and confers pure antiestrogenic activity

Fanning, S.W. and Hodges-Gallagher, L. et al.

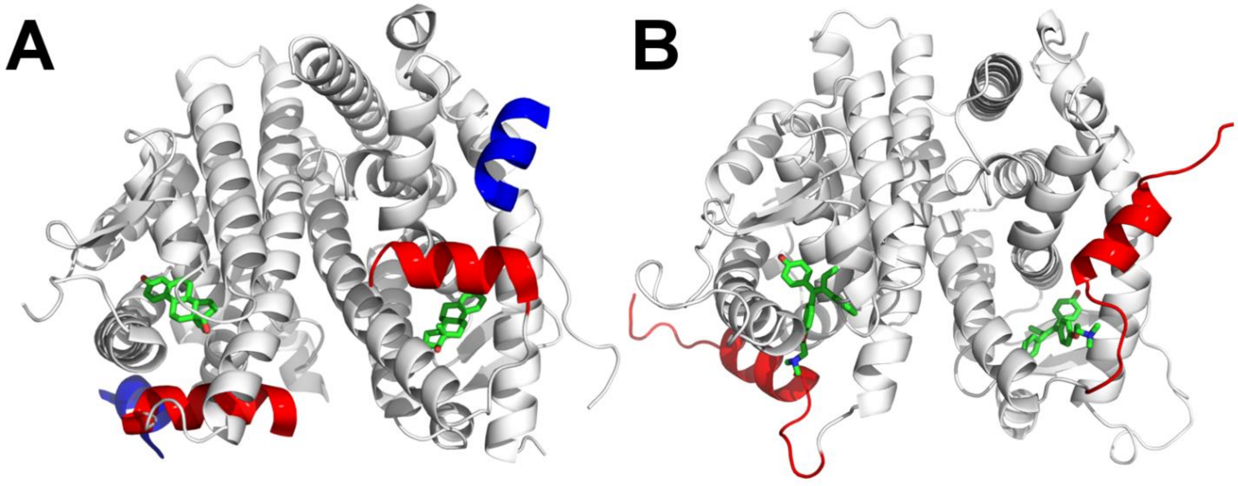

Supplementary Figure 1. Helix 12 conformational differences in the agonist-bound (E2, **A**) and SERM-bound (4-OHT, **B**) states. Helix 12 is highlighted in red. E2 or 4-OHT are shown as green sticks. TIF2 NRBOX3 (coregulator) peptide is highlighted in blue. Protein data bank (PDB) sequences used are 1GWR (A) and 3ERT (B).

Supplementary Figure 2: Specificity of OP-1074

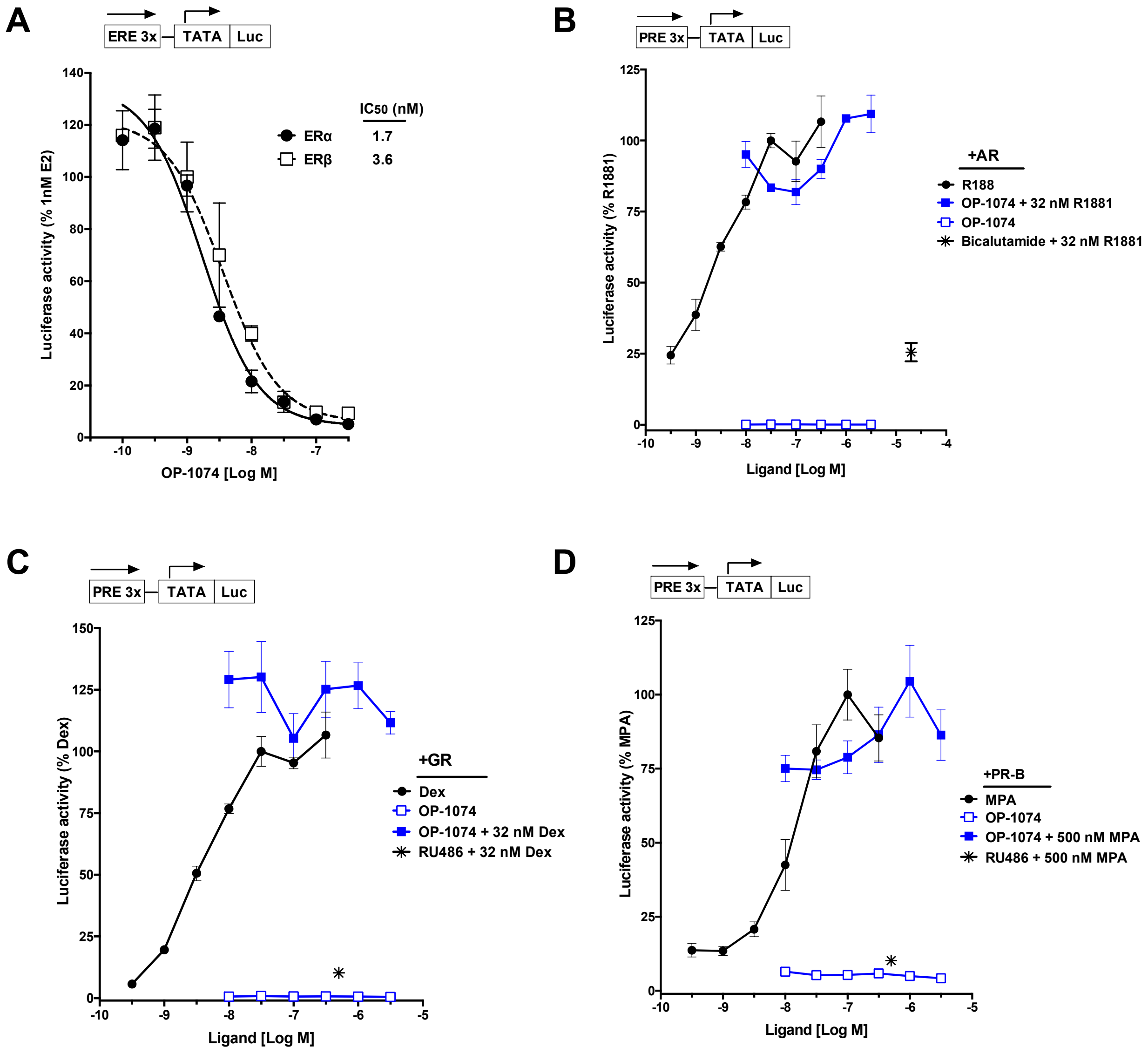

Supplementary Figure 2. OP-1074 is specific for ERα and ERβ. **A**, OP-1074 inhibits E2-stimulated ERα and ERβ mediated transcription. HeLa cells were transfected with an estrogen responsive reporter gene, ERE3X-TATA-Luc, along with wild type ERα or ERβ, and treated with OP-1074 in the presence of 1 nM E2 for 22 hours. Shown is the mean luciferase activity

normalized to percent of 1 nM E2 alone with standard error from triplicate wells of one representative experiment. **B-D**, OP-1074 lacks agonist and antagonist activity on androgen receptor (AR, (**B**)), glucocorticoid receptor (GR, (**C**)), and the B-isoform of the progesterone receptor (PR-B, (**D**)). HeLa cells were transfected with the designated wild type receptor along with reporter gene PRE3X-TATA-Luc, which is promiscuously activated with known agonists specific for each receptor: R1881 for AR, dexamethasone (Dex) for GR, and medroxyprogesterone acetate (MPA) for PR-B. Each receptor was also dosed with one concentration of known antagonist for each receptor: 32 nM bicalutamide for AR, and 500 nM RU486 for GR and PR. Transfected cells were treated with the designated ligands for 22 hours and luciferase activity assayed. Shown are representative experiments, independently performed at least 3 times. Note that curves for agonists for each receptor were performed in separate experiments. Shown are means with SEM, normalized to percent of 32 nM R1881 for AR, 32 nM Dex for GR, and 0.5 µM MPA for PR-B (except with MPA alone, which was normalized to 1 nM MPA).

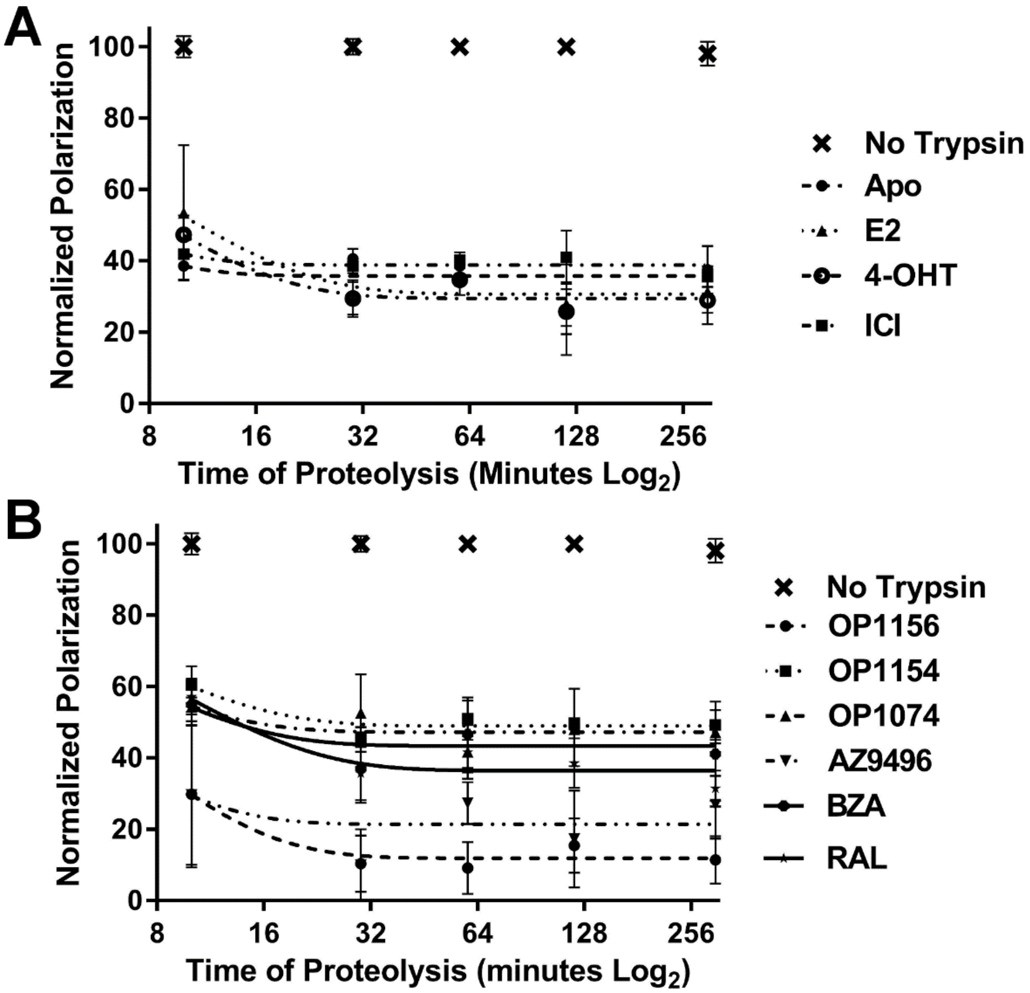

Supplementary Figure 3: Trypsin-coupled fluorescence polarization assay of ERα LBD helix 12 mobility. **A,** Log_2 timescale from a representative experiment highlighting differences in half-lives ($t_{1/2}$) between the apo, E2, 4-OHtamoxifen and fulvestrant-bound states. Each point represents the mean polarization normalized to non-trypsin control +/- SEM from triplicate samples. Curves were fit to an exponential decay model with $R_2 \geq 0.9$. **B**, Log_2 timescale assayed as above highlighting differences in $t_{1/2}$ between raloxifene, baxedoxifene, and OP-1074 analogs.

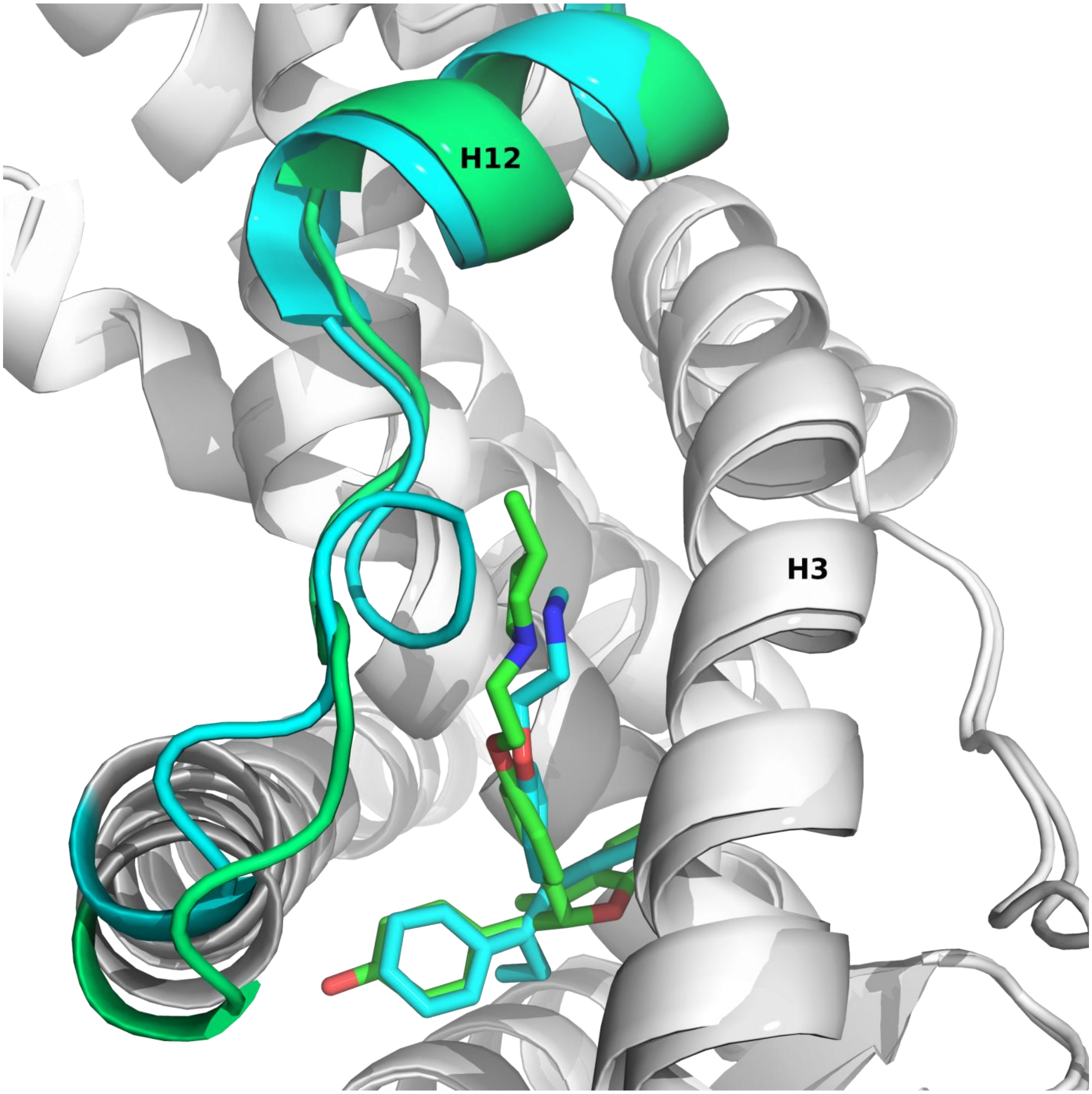

Supplementary Figure 4. Superposition of ERα LBD-OP-1074 (green) and endoxifen (cyan)-bound x-ray crystal structures. Endoxifen was chosen because it uses the same protein construct as the OP-1074 structure. PDB IDs: 5W9D and 5UFX.

Supplementary Table 1. Crystallographic data collection and refinement statistics.

	**ERα LBD OP-1156**	**ERα LBD OP-1154**	**ERα LBD OP-1074**
PDB ID	6C42	5UFW	5UFX
**Data collection**			
Space group	C2	C2	C2
a, b, c (Å)	102.81, 58.01, 87.85	102.57, 57.88, 87.70	102.72, 58.02, 87.83
α, β, γ	90, 103.013, 90	90, 102.91, 90	90, 102.90, 90
Resolution Range	20.00 – 2.00 Å	20.00 – 1.58	20 – 1.55
Number of Reflections (all/unique)	34199/9242	68281/18966	70195/19498
I/σ (highest resolution)	2.0	1.9	1.9
R_{merge} (highest resolution)	7.80 (46.2)	6.55 (49.1)	4.7 (47.2)
Completeness (highest resolution)	99.6 (100)	99.7 (96.0)	96.2 (99.9)
Redundancy	3.7	3.6	3.6

**Refinement**			
R_{work}/R_{free}	22.83/26.84	17.97/21.04	19.52/22.99
No. Atoms	4102	4502	4417
Water Molecules	471	623	618
Ligand Molecules	2	2	2
Bond lengths (Å)	0.002	0.005	0.007
Bond angles (°)	0.555	0.877	1.121
**Ramachandaran plot statistics**			
Preferred number (%)	431 (97.73)	416 (98.58)	418 (98.12)
Additional allowed (%)	10 (2.27)	6 (1.42)	8 (1.88)
Outliers (%)	0	0	0

Supplementary Methods

ER-specific luciferase reporter gene (related to Supplementary Figure 1)

45,000 HeLa cells per well were transiently transfected using manufacturer's protocol for Lipofectamine LTX (ThermoFisher). HeLa cells were transfected with 3 ng of plasmids for

either ERα or ERβ, and 85 ng ERE-3x-Luc, containing 3 copies of estrogen-response element (ERE) cloned into pGL4.23 luciferase reporter gene containing a minimal TATA promoter (Promega). Transfected cells were treated with OP-1074 in the presence of 100 pM E2 for 22 hours (1.25% total final charcoal dextran stripped FBS). Bright Glo luciferase reagent (Promega) was used to lyse cells and detect firefly luciferase activity, measured in relative light units, at 1 sec intervals using the Varioskan Lux multimodal plate reader. To rule out non-specific nuclear receptor activity, HeLa cells were transfected as above but also with plasmids for full length wild type glucocorticoid receptor (GR), progesterone receptor (PR), or androgen receptor (AR), along with PRE-3x-Luc, containing 3 copies of progesterone response element (PRE) cloned into pGL4.23 luciferase reporter gene containing a minimal TATA promoter. Transfected cells were treated with OP-1074, and/or designated agonists and antagonists. R1881, bicalutamide, dexamethasone, medroxyprogesterone acetate and RU486 were purchased from Sigma Aldrich. Data shown in figures are representative experiments reproduced at least 3 times in independent luciferase assays

Synthesis of OP-1074, OP-1154, OP-1156, OP-1039, OP-1047

<u>**1-(2,4-Dihydroxyphenyl)-2-(4-hydroxyphenyl)ethanone**</u>

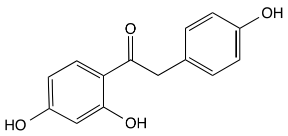

Resorcinol (1,3-dihydroxybenzene, 200 g, 1.82 mol, 1.0 equiv.) and 4-hydroxyphenylacetic acid (304 g, 2.0 mol, 1.1 equiv.) were added to a 3 neck 5 L round bottomed flask fitted with a magnetic stir bar, a pressure equalizing addition funnel, thermometer and heating mantle. Toluene (890 mL) was added to the flask to give a suspension. The reaction flask was then purged with nitrogen and the addition funnel filled with boron trifluoride etherate (639 mL, 5.09 mol, 2.8 equiv.). The reaction was heated and stirred. Boron trifluoride etherate was added dropwise over several minutes. The reaction went through various changes in color from yellow to dark red during the addition of the Lewis acid. After the addition of boron trifluoride etherate was complete, the addition funnel was removed and replaced with a condenser and the reaction vessel was warmed with the heating mantle. When the reaction temperature reached 90°C, the suspension slowly dissolved, to form a dark homogenous solution briefly before a yellow precipitate formed. TLC (50% EA/Hex) indicated the reaction was complete. The reaction was cooled to ambient temperature in an ice-bath. The slurry was poured into a 12 % aqueous solution of sodium acetate (144 g, 1.2 L) at 0°C and stirred at room temperature overnight. The light yellow solid was filtered and washed with water (2 x) and a small amount of diethyl ether (1 x). The cream solid was air-dried for 4 days (362 g, 76.7 %, HPLC 94 %, m.p. 186-188 °C). (lit. m.p: 180 °C, Reference: JOC, 2000, 65, 2305).

1-(2-hydroxy-4-((tetrahydro-2H-pyran-2-yl)oxy)phenyl)-2-(4-((tetrahydro-2H-pyran-2-yl)oxy)phenyl)ethanone

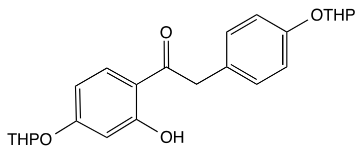

1-(2,4-Dihydroxyphenyl)-2-(4-hydroxyphenyl)ethanone (186.0 g, 0.76 mol, 1.0 equiv.) and ethyl acetate (600 mL) were added to a three neck round bottomed flask (5 L) equipped with a magnetic stir bar, a thermometer and a nitrogen inlet. The flask was evacuated and then repressurized with nitrogen. 3,4-dihydro-2H-pyran (312.6 mL, 3.43 mol, 4.5 equiv.) was then added via graduated cylinder under positive nitrogen pressure in the reaction vessle. p-toluenesulfonic acid (155 mg, 0.9 mmol, 0.001 equiv.) was added to the reaction mixture. The reaction vessel was placed in an acetone bath (20 ^{o}C). Over 1 h, the temperature increased to 45 ^{o}C At that time, additional p-Toluenesulfonic acid (310 mg, 1.8 mmol, 0.002 equiv.) was added. The reaction suspension became an orange solution. The solution was stirred at room temperature overnight, over which time thick suspension formed. Triethylamine (15 mL) was added to the mixture. The mixture was then concentrated under reduced pressure. The resulting residue was crystallized from 2-propanol. The white solid was collected by filtration and dried in a vacuum oven at ~40 ^{o}C overnight (150 g, 44 %, HPLC 92 %, m.p. 112-115 °C). (lit. m.p.: 118 °C, J. Med. Chem., 1990, 33, 3322),

2-(4-iodophenyl)-7-((tetrahydro-2H-pyran-2-yl)oxy)-3-(4-((tetrahydro-2H-pyran-2-yl)oxy)phenyl)chroman-4-one

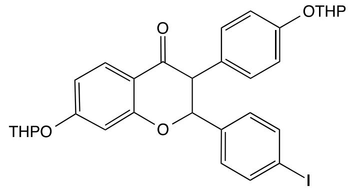

1-(2-Hydroxy-4-((tetrahydro-2H-pyran-2-yl)oxy)phenyl)-2-(4-((tetrahydro-2H-pyran-2-yl)oxy)phenyl)ethanone (293.0 g, 0.71 mol, 1.0 equiv.) was added to a 3 neck 5 L round

bottomed flask. 2-butanol (1.25 L) and 97.0% 4-iodobenzaldehyde (169.9 g, 0.71 mol, 1.0 equiv.) were added to the flask to give a suspension. Piperidine (23.5 mL, 0.24 mol, 0.3 equiv.) and 1, 8-diazabicyclo[5.4.0]undec-7-ene (36.4 mL, 0.24 mol, 0.3 equiv.) were added to the suspension. The flask was fitted with a Dean-Stark apparatus, condenser, thermometer with an inlet adapter, and magnetic stir bar. The reaction was stirred and heated under a nitrogen atmosphere with a mantle to give an orange solution. The solution temperature was held at 78 ^{o}C. Half of the solvent volume (610 mL) was collected over 1.5 hours. The reaction was heated for another hour without collecting additional 2-butanol. The solution gradually darkened to a red color. The mantle was removed and the flask was allowed to cool, at which point 2-propanol (500 mL) was added and the resulting mixture stirred. A large mass formed at the bottom of the flask when the temperature dropped below 50 °C. The reaction was left to stir for 2 days. The flask contained a solid cake and a clear solution. The supernatant was decanted and the solid was mixed with 2-propanol (100 mL) and diethyl ether (80 mL) and slowly warmed with agitation using a metal spatula until the mixture could freely stir with a stir bar. The solid mass became a viscous suspension. 25% diethyl ether/hexanes (300 mL) was added to the mixture and the suspension stirred for 1 h. The suspension was filtered and the solid washed with 25% diethyl ether/hexanes to give a white powder (240 g, 54 %). The filtrate was concentrated and recrystallization was performed again as above to give a second crop of solid product (40 g, 9 %). The filtrates and the supernatant were combined, concentrated, adsorbed onto silica gel and purified on silica gel with 30 % ethyl acetate in hexanes to give a yellow foam (137 g, 31 %, m.p. 136-138 °C). Overall yield 94%. ^{1}H NMR was consistent with published spectral data (WO2011/156518). ^{1}H NMR, (300 MHz, $CDCl_3$) δ 7.9 (dd, j = 2.1, 9, 1H), 7.6 (d, j

= 8.1, 2H), 7.25-6.70 (m, 8H), 5.49-5.32 (m, 3H), 4.02-3.96 (m, 1H), 3.91-3.82 (m, 2H), 3.66-3.56 (m, 2H), 2.06-1.95 (m, 2H), 1.95-1.81 (m, 4H), 1.72-1.62 (m, 6H).

<u>2-(4-iodophenyl)-4-methyl-7-((tetrahydro-2H-pyran-2-yl)oxy)-3-(4-((tetrahydro-2H-pyran-2-yl)oxy)phenyl)chroman-4-ol</u>

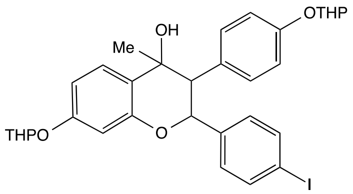

To a solution of 90.0 % 2-(4-iodophenyl)-7-((tetrahydro-2H-pyran-2-yl)oxy)-3-(4-((tetrahydro-2H-pyran-2-yl)oxy)phenyl)chroman-4-one (234.0 g, 0.34 mol, 1.0 equiv.) in tetrahydrofuran (0.89 L) at -5°C, was added methylmagnesium chloride (3.0 M solution in THF, 200 mL, 0.60 mol, 1.8 equiv.) by addition funnel over 30 minutes. The temperature was kept below 0 °C during the addition. After addition of the Grignard reagent was complete, the reaction was removed from the ice bath, allowed to reach room temperature, and stirred at room temperature overnight. TLC (20 % EA/Hex) indicated that the reaction was complete. The reaction was cooled to 0 °C and carefully quenched with saturated ammonium chloride (100 mL), the temperature was kept below 7° C during the quench. The mixture was stirred at room temperature for 30 min before the solid was filtered through a bed of Celite. The solid was rinsed with DCM twice and the filtrate was dried over anhydrous sodium sulfate, filtered and concentrated. The resulting pale yellow foam was used directly in the next reaction without purification.

3-(4-Hydroxyphenyl)-2-(4-iodophenyl)-4-methyl-2H-chromen-7-ol

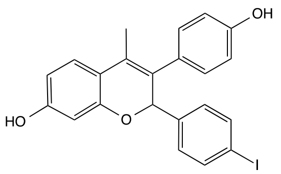

2-(4-Iodophenyl)-4-methyl-7-((tetrahydro-2H-pyran-2-yl)oxy)-3-(4-((tetrahydro-2H-pyran-2-yl)oxy)phenyl)chroman-4-ol (17.89 g, 27.8 mmol) and 80 % acetic acid in H_2O (120 mL) was added to a 1 L RB flask. The flask was degassed, flushed with nitrogen and heated at 90 ^oC for 1 hour. HPLC analysis of the reaction showed no starting material was present. The solvent was removed to give a red oil. The red oil was dissolved into ethyl acetate (200 mL) and washed with saturated sodium bicarbonate solution (4 x 100 mL). The combined aqueous layer was extracted with ethyl acetate (100 mL). The organic layers were combined and washed with brine (100mL), filtered and concentrated to give a red oil (18.26 g, crude). The oil was loaded onto 55 g of silica gel and chromatographed on silica gel (2 x 100 g cartridge). Fractions containing the product were pooled and concentrated to a light red oil. The oil was repeatedly dissolved into DCM and concentrated until a light red foam formed. The foam was suspended into DCM and filtered to give a pink solid (9.4036 g, 74.0 %). ^1H NMR, (300 MHz, DMSO, D_6) δ 9.60 (d, j = 9.3, 2H), 7.58 (d, j = 8.1, 2H), 7.09 (d, j = 8.4 2H), 7.05 (dd, j = 2.1, 9.0, 2H), 6.7 (d, j = 8.4, 2H), 6.3 (dd, j = 2.4, 9.0, 1H), 6.1 (d, j = 2.1, 1H), 5.9 (s, 1H), 3.3 (s, 1H), 2.0 (s, 3H).

2-(4-iodophenyl)-4-methyl-7-((tetrahydro-2H-pyran-2-yl)oxy)-3-(4-((tetrahydro-2H-pyran-2-yl)oxy)phenyl)-2H-chromene

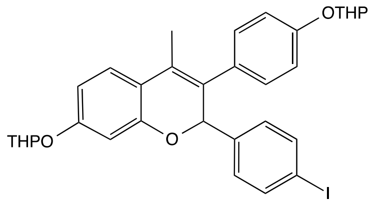

To a solution of 3-(4-hydroxyphenyl)-2-(4-iodophenyl)-4-methyl-2H-chromen-7-ol (150.0 g, 0.33 mmol, 1.0 equiv.) and pyridinium p-toluene sulfonate (16.46 g, 65.8 mmol, 0.2 equiv.) in DCM (300 mL) was added 3,4-dihydro-2H-pyran (165 mL, 1.81 mol, 5.5 equiv.). The reaction was stirred at room temperature for 2 days. Aqueous saturated sodium bicarbonate was added to the reaction and stirred for 1 h and the layers separated. The DCM layer was washed with sodium bicarbonate again, water (2 x), brine, dried over anhydrous magnesium sulfate, filtered and concentrated. The resulting viscous oil was triturated with IPA and hexanes. The suspension was stirred overnight and the solid was filtered and washed with cold IPA. The resulting off-white powder was dried in air (154 g, 75 %, m.p. 153-155 °C). ^{1}H NMR, (300 MHz, $CDCl_3$) δ 7.53 (d, j = 8.7, 2H), 7.17 (d, j = 8.4, 1H), 7.056.95 (m, 6H), 6.62 (ddd, J = 2.4, 5.7, 11.1, 1H), 6.49 (dd, j = 2.4, 5.4, 1H), 5.85 (s, 1H), 5.40-3.34 (m, 2H), 3.91-3.87 (m, 2H), 3.63-3.57 (m, 2H), 2.07 (s, 3H), 2.03-1.60 (m, 12H).

(3R)-3-Methyl-1-(2-(4-(4-methyl-7-((tetrahydro-2H-pyran-2-yl)oxy)-3-(4-((tetrahydro-2H-pyran-2-yl)oxy)phenyl)-2H-chromen-2-yl)phenoxy)ethyl)pyrrolidine

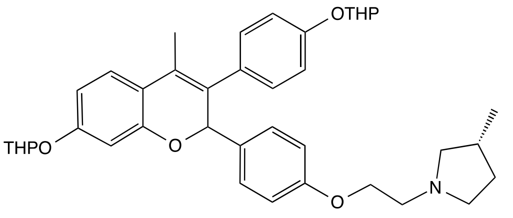

(R)-3-Methylpyrrolidine hydrochloride (127.94 g, 1.05 mol, 1.0 equiv.), acetonitrile (2.0 mL) and potassium carbonate (290.8 g, 2.1 mol, 2.0 equiv.) were placed into a three neck 5 L round bottomed flask fitted with a condenser, overhead stirrer with a water cooled collar and a pressure equalizing funnel. The reaction was heated to reflux with a heating mantle. 2-chloroethanol (72.0 mL, 1.07 mol, 1.0 equiv.) dissolved in acetonitrile (500 mL) was slowly added dropwise to the refluxing reaction mixture. The reaction was heated at reflux for 16 h. The reaction was then filtered, and the solid washed with acetonitrile (500 mL). Additional powdered potassium carbonate (290 g, 2. equiv) was added to the reaction. The reaction was again heated at reflux for another 16 h. The reaction mixture was then allowed to cool to room temperature, filtered, and the solids washed with ethyl acetate (2 x 500 mL). The filtrate was concentrated to a yellow liquid. The liquid was vacuum distilled through a short path distillation apparatus. The desired amino alcohol was collected at 54 °C, 0.025 torr. as a colorless liquid (78.4 g, 57.7 %).

A 1L round bottom flask was evacuated and backfilled with argon (3X) and then charged with 2-(4-iodophenyl)-4-methyl-7-((tetrahydro-2H-pyran-2-yl)oxy)-3-(4-((tetrahydro-2H-pyran-2-yl)oxy)phenyl)-2H-chromene (126.0 g, 0.202 mol, 1.0 equiv.), (R)-2-(3-methylpyrrolidin-1-yl)ethanol (78.12 g, 0.61 mol, 3.0 equiv.), 1,10-Phenanthroline (7.27 g, 40.4 mmol, 0.2 equiv.), and Cesium carbonate (131.47 g, 0.404 mol, 2.0 equiv.) in butyronitrile (500 mL). The suspension was tan in color. Copper(I) iodide (38.42 g, 0.202 mol, 1.0 equiv.) was added to the suspension and the flask was evacuated and backfilled with argon (3 x). Addition of CuI changed the color to red brown. The flask was sealed with a rubber septum and held in place with a hose clamp. The reaction mixture was heated in an oil bath at 120 °C. After heating for 64 hours, the dark brown suspension was cooled to room temperature and the mixture filtered through a pad of Celite (3 cm). The solid was washed with DCM (600 mL). The filtrate was collected and

concentrated. The residue was adsorbed onto silica gel (200 g) and purified with silica gel (4 x 100 g cartridge, 5-25 % EA/Hexanes, then 0 - 40 % MeOH/DCM) [TLC: 5 % MeOH/DCM. The product was isolated as a yellow foam (75.09 g, 59.4 %). ^{1}H NMR, (300 MHz, $CDCl_3$) δ 7.23-7.16 (m, 3H), 7.07-7.03 (m, 2H), 6.97-6.94 (m, 2H), 6.76-6.73 (m, 2H), 6.61 (ddd, j = 2.4, 6.0, 8.7, 2H), 6.47 (dd j = 2.4, 5.7, 1H), 5.83 (s, 1H), 5.40-5.30 (m, 2H), 4.04 (dd, j = 3, 3 2H), 3.94-3.83 (m, 2H), 3.63-3.52 (m, 2H), 2.98-2.77 (m, 3H), 2.59-2.50 (m, 1H), 2.32-2.19 (m, 1H), 2.12-1.75 (m), 1.70-1.52 (m), 1.41-1.33 (m, 1H), 1.11-1.03 (m, 3H).

(3R)-1-(2-(4-(7-hydroxy-3-(4-hydroxyphenyl)-4-methyl-2H-chromen-2-yl)phenoxy)ethyl)-3-methylpyrrolidin-1-ium acetate (OP-1038)

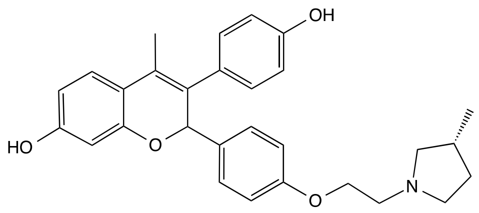

(3R)-3-Methyl-1-(2-(4-(4-methyl-7-((tetrahydro-2H-pyran-2-yl)oxy)-3-(4-((tetrahydro-2H-pyran-2-yl)oxy)phenyl)-2H-chromen-2-yl)phenoxy)ethyl)pyrrolidine (225.25 g, 0.360 mol, 1.0 equiv.) was dissolved into 80 % acetic acid/water (2.2 L, previously degassed with argon). The solution was heated in an oil bath at 90 °C for 1 hour under an argon atmosphere. HPLC analysis of the reaction mixture indicated the reaction was complete. The dark red solution was concentrated to a dark red oil *in vacuo* with the bath temperature set at or below 55 °C. The low pressure was released by backfilling with nitrogen atmosphere. The resulting oil was dissolved in methanol (1.8 L) and the solution brought to pH 7, with 2 N sodium hydroxide solution and

brought to pH 8 with saturated $NaHCO_3$. The dark red heterogeneous liquid was concentrated to a dark red oil suspended in an aqueous mixture. The thick oil was allowed to settle and harden for 15 minutes. The aqueous layer was decanted and the residue rinsed with water (3 x 600 mL). The combined aqueous layer was extracted with ethyl acetate (2 x 800 mL). The combined organic layer was washed with brine (2 x 400 mL), dried over anhydrous magnesium sulfate, filtered and the filtrate mixed with the red residue and concentrated to give a dark red residue. The residue was adsorbed onto silica gel (375 g) and chromatographed on silica gel (7 x 120 g cartridge) with 0-30 % MeOH in DCM. The product was a red colored foam (92.01 g, 49.4 %). ^{1}H NMR, (300 MHz, $DOCD_3$) δ 7.20 (dd, j = 2.1, 6.6, 2H), 7.11 (d, j= 8.4, 1H), 6.98 (dd, j = 2.1, 6.3, 2H), 6.78 (dd, j = 2.1, 6.6, 2H), 6.70 (dd, j = 2.1, 6.6, 2H), 6.34 (dd, j = 2.7, 8.4, 1H), 6.11 (d, j = 2.4, 1H), 5.78 (s, 1H), 4.07 (t, j = 5.0, 2H), 3.1-2.7 (brm, 4H), 2.37-2.33 (m, 1H), 2.14-2.03 (m, 1H), 2.04 (s, 3H), 1.5-1.38 (m, 1H), 1.06 (d, j = 6.3, 3H).

2-(4-hydroxyphenyl)-7-((tetrahydro-2*H*-pyran-2-yl)oxy)-3-(4-((tetrahydro-2*H*-pyran-2-yl)oxy)phenyl)chroman-4-one

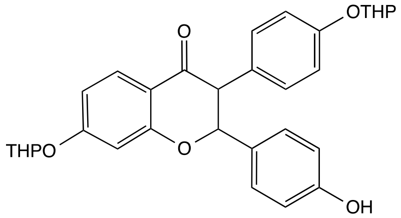

A solution of 1-(2-hydroxy-4-((tetrahydro-2H-pyran-2-yl)oxy)phenyl)-2-(4-((tetrahydro-2H-pyran-2-yl)oxy)phenyl)ethanone (6 g, 0.015 mol), 4-hydroxybenzaldehyde (1.59 g, 0.013 mol) and piperidine (370 mg, 4.0 mmol) in benzene (100 mL) was refluxed using Dean-Stark apparatus for 16 h. The progress of the reaction was monitored by TLC using 25 % v/v ethyl acetate in petroleum ether as eluting solvent. After completion of reaction, the reaction mixture

was cooled to room temperature and the solvent was removed under reduced pressure to get crude product. The crude product was purified by flash column chromatography (silica gel, 25% v/v ethyl acetate in petroleum ether) to obtain 2-(4-hydroxyphenyl)-7-((tetrahydro-2*H*-pyran-2-yl)oxy)-3-(4-((tetrahydro-2*H*-pyran-2-yl)oxy)phenyl)chroman-4-one as white solid (1.6 g, 21 %). ^{1}H NMR, (300 MHz, DMSO D_6) δ 9.46 (s, 1H), 7.74 (d, j = 8.7, 1H), 7.20 (d, j = 7.8, 2H), 6.98 (d, j = 7.8, 2H), 6.82-6.61 (m, 6H), 5.73 (t, j = 10.2 1H), 5.61 (s, 1H), 5.36 (s, 1H), 4.49 (d, j = 12, 1H), 3.81-3.65 (m, 2H), 3.65-3.43 (m, 2H), 1.85-1.22 (brm, 12H).

(*R*)-2-(2-methylpyrrolidin-1-yl)ethan-1-ol

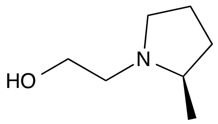

To a solution of (R)-2-methyl pyrrolidine hydrochloride (0.5 g, 4.11 mmol) in acetonitrile (10 mL) was added K_2CO_3 (1.72 g, 12.3 mmol) and 2-bromoethanol (0.5 g, 4.1 mmol). The reaction mixture was heated at 80 °C for 1 h. After completion of reaction (by TLC), the reaction mixture was diluted with acetonitrile and filtered. The filtrate was concentrated under vacuum to afford the product (330 mg, crude) as yellow oil. The product formation was confirmed by LCMS (M+1: $C_7H_{16}NO$ calc'd.= 130.12, obs. = 130.1).

(*R*)-1-(2-chloroethyl)-2-methylpyrrolidine

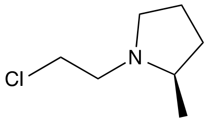

To a solution of (*R*)-2-(2-methylpyrrolidin-1-yl)ethan-1-ol (0.3 g, 2.3 mmol) in 1, 2-dichloroethane (10 mL), thionyl chloride (0.20 mL, 2.7 mmol) was added drop wise at 0 ^{o}C. The reaction mixture was then gradually heated to 80 ^{o}C and held at that temperature for 2 h. The excess solvent and thionyl chloride were removed under reduced pressure to furnish crude (*R*)-1-(2-chloroethyl)-2-methylpyrrolidine (0.4 g, 93%). This material was taken forward without further purification into next step.

2-(4-(2-((*R*)-2-methylpyrrolidin-1-yl)ethoxy)phenyl)-7-((tetrahydro-2*H*-pyran-2-yl)oxy)-3-(4-((tetrahydro-2*H*-pyran-2-yl)oxy)phenyl)chroman-4-one

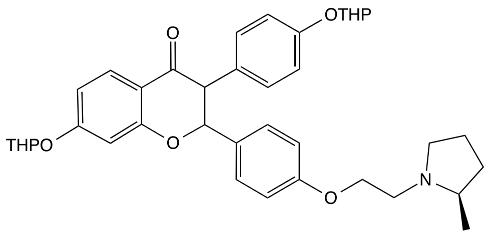

To a stirred solution of 2-(4-hydroxyphenyl)-7-((tetrahydro-2*H*-pyran-2-yl)oxy)-3-(4-((tetrahydro-2*H*-pyran-2-yl)oxy)phenyl)chroman-4-one (1 g, 1.93 mmol) in dry acetone (10 mL) was added cesium carbonate (1.82 g, 5.8 mmol) at 0 ^{o}C and the reaction mixture was stirred for 10 min. Then a solution of (R)-1-(2-chloro-ethyl)-2-methyl-pyrrolidine hydrochloride (0.34 g, 2.30 mmol) in 2 mL dry acetone was slowly added at 0°C After completion of addition, the reaction mixture was gradually heated to reflux and maintained for 18 h. Reaction was monitored by TLC (using 20% methanol in ethyl acetate as eluting solvent). After completion of reaction, the reaction mixture was filtered, washed with acetone and concentrated to get 2-(4-(2-((*R*)-2-methylpyrrolidin-1-yl)ethoxy)phenyl)-7-((tetrahydro-2*H*-pyran-2-yl)oxy)-3-(4-((tetrahydro-2*H*-pyran-2-yl)oxy)phenyl)chroman-4-one as yellow oil. The product formation was confirmed by

LCMS (M+1, $C_{38}H_{46}NO7$, calc'd = 628.33, obs. 628.4) This crude product was taken forward in the next step.

3-(4-hydroxyphenyl)-4-methyl-2-(4-(2-((*R*)-2-methylpyrrolidin-1-yl)ethoxy)phenyl)-2*H*-chromen-7-ol (OP-1039)

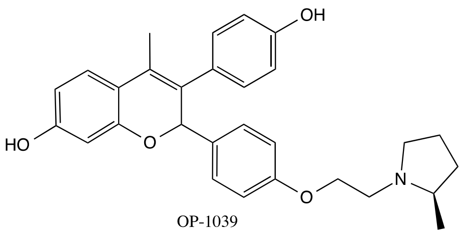

OP-1039

To a cooled (0 °C) solution of crude 2-(4-(2-((*R*)-2-methylpyrrolidin-1-yl)ethoxy)phenyl)-7-((tetrahydro-2*H*-pyran-2-yl)oxy)-3-(4-((tetrahydro-2*H*-pyran-2-yl)oxy)phenyl)chroman-4-one (1 g, 1.59 mmol obtained from the previous step) in dry THF (5 mL), CH_3MgI (5.4 mL, 8.1 mmol 1.5 M solution in THF) was added drop wise. After completion of addition, the reaction mixture was slowly allowed to reach room temperature and stirred for 6 h. Then reaction mixture was cooled to 0 ^{0}C, quenched with ammonium chloride solution (10 mL) and extracted with ethyl acetate (3 x 10 mL). The combined organic layer was dried over anhydrous sodium sulfate, filtered and concentrated under vacuum to get the methyl adduct as yellow oil. The crude oil was taken in acetic acid (9 mL) and water (1 mL) and heated at 90 ^{0}C for 2 h. The reaction mixture was cooled to room temperature and concentrated under vacuum. The residue obtained was taken up in EtOAc and quenched with saturated $NaHCO_3$ solution at 0 °C. The organic layer was separated and aqueous layer extracted with EtOAc (2 x 10 mL). The combined organic layers were dried over anhydrous sodium sulfate, filtered and concentrated to obtain the crude product

as a viscous oil. The crude product was purified by flash column chromatography (silica gel, 25% v/v ethyl acetate in petroleum ether) followed by preparative HPLC to furnish OP-1039 as beige colored solid (80 mg, 12 %) ^{1}H NMR, (300 MHz, $DOCD_3$) δ 7.21 (d, J = 8.7, 2H), 7.14 (d, j = 8.4, 1H), 7.00 (d, j = 8.7, 2H), 6.79 (d, j = 8.7, 2H), 6.72 (d, j = 8.4, 2H), 6.36 (dd, j = 2.4, 8.4, 1H), 6.13 (d, j = 2.4, 1H), 5.80 (s, 1H), 4.11-4.6 (m, 2H), 3.45-3.15 (m, 2H), 2.60-2.43 (m, 2H), 2.33 (q, j = 9.3, 1H), 2.05 (s, 3H), 2.05-1.90 (m, 1H), 1.85-1.71 (m, 2H), 1.51-1.38 (m, 1H), 1.15 (d, j = 6, 3H).

2-(4-(2-(pyrrolidin-1-yl)ethoxy)phenyl)-7-((tetrahydro-2*H*-pyran-2-yl)oxy)-3-(4-((tetrahydro-2*H*-pyran-2-yl)oxy)phenyl)chroman-4-one

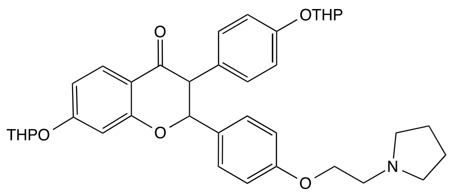

To the stirred solution of 2-(4-hydroxyphenyl)-7-((tetrahydro-2*H*-pyran-2-yl)oxy)-3-(4-((tetrahydro-2*H*-pyran-2-yl)oxy)phenyl)chroman-4-one (1g, 1.9 mmol) in dry acetone (10 vol), was added cesium carbonate (1.8 g, 5.8 mmol) at 0 °C and stirred for 10 min. Then a solution of 1-(2-chloro-ethyl)-pyrrolidine (0.5 g, 2.9 mmol) in acetone (2 mL) was added. After completion of addition, the reaction mixture was gradually heated to reflux and maintained for 18 h. The reaction mixture was filtered, washed with acetone and concentrated to provide 2-(4-(2-(pyrrolidin-1-yl)ethoxy)phenyl)-7-((tetrahydro-2*H*-pyran-2-yl)oxy)-3-(4-((tetrahydro-2*H*-pyran-2-yl)oxy)phenyl)chroman-4-one as yellow oil (1g, 84%), which was used as such for next step.

<u>3-(4-hydroxyphenyl)-4-methyl-2-(4-(2-(pyrrolidin-1-yl)ethoxy)phenyl)-2*H*-chromen-7-ol (OP-1040)</u>

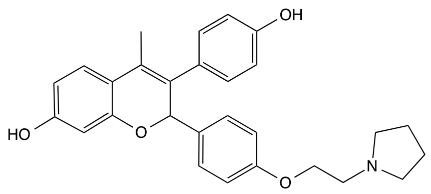

OP-1040

To a cooled (0 °C) solution of 2-(4-(2-(pyrrolidin-1-yl)ethoxy)phenyl)-7-((tetrahydro-2*H*-pyran-2-yl)oxy)-3-(4-((tetrahydro-2*H*-pyran-2-yl)oxy)phenyl)chroman-4-one (1g, 1.6 mmol) in dry THF (5 mL) CH_3MgI (5.4 mL, 8.1 mmol, 1.5 M solution in THF) was added drop wise at 0 °C. After completion of addition, the reaction mixture was slowly allowed to reach room temperature and stirred for 6 h. Then reaction mixture was cooled to 0 ^{0}C, quenched with ammonium chloride solution (10 mL) and extracted with EtOAc (3 x 10 mL). The combined organic layer was dried over anhydrous sodium sulphate, filtered and concentrated under vacuum to isolate the crude methyl adduct as yellow oil. The oil was taken in acetic acid (9 mL), water (1 mL) and heated to 90 ^{0}C for 2 h. After completion of reaction, the reaction mixture was cooled to room temperature and concentrated under vacuum to remove the solvents. The residue was taken in EtOAc and quenched with saturated $NaHCO_3$ solution at 0°C. The organic layer was separated and aqueous layer extracted with EtOAc (2 x 10 mL). The combined organic layer was dried over anhydrous sodium sulphate, filtered and concentrated to get the crude product. The crude product was purified by flash column chromatography (silica gel, EtOAc / Pet ether) followed by preparative HPLC to get pure 3-(4-hydroxyphenyl)-4-methyl-2-(4-(2-(pyrrolidin-1-yl)ethoxy)phenyl)-2*H*-chromen-7-ol (OP-1040) (70 mg, 10%), as beige colored solid. The product was confirmed by LCMS (M+1 $C_{28}H_{30}NO_4$, calc'd = 444.21, obs. = 444.2).

(*R*)-1-(2-(benzyloxy)ethyl)-2-methylpyrrolidine

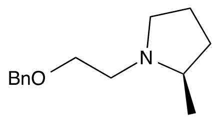

(S)-3-Methylpyrrolidine hydrochloride (0.0524 g, 0.428 mmol, 1.07 equiv), 2-benzyloxyacetaldehyde (0.0598 g, 0.4 mmol), and sodium triacetoxyborohydride (0.1823g, 0.859 mmol, 2.15 equiv) and THF (4 mL) was added to a 20 mL scintillation vial under a stream of nitrogen to give a colorless suspension. The vial was sealed, fitted with a drying tube and stirred at room temperature for 16 h. The reaction remained a colorless suspension. The vigorously stirred reaction was quenched with saturated sodium bicarbonate (5 mL) for 10 minutes. The mixture was poured into a separatory funnel and the layers separated. The aqueous layer was extracted with ethyl acetate (3 x 10 mL) and the organic layers combined, dried over anhydrous magnesium sulfate, filtered and concentrated to a colorless oil (103 mg). The oil was loaded onto 0.4 g of silica gel and chromatographed on a 4 g silica gel cartridge. 0-40 %MeOH in DCM. The desired fractions were pooled and concentrated to give a colorless oil (0.0544g, 61.6 %) ^{1}H NMR, (300 MHz, $CDCl_3$) δ 7.34-7.25 (m, 5H), 4.53 (s, 2H), 3.64 (t, j = 5.7, 2H), 3.05 (dd, j = 7.5, 9.6, 1H), 2.91-2.70 (m, 3H), 2.66 (dt, j = 6.0, 9.0, 1H), 2.37-2.22 (m, 1H), 2.18 (dd, j = 8.1, 9.3, 1H), 1.98 (brs, 1H), 1.46-1.34 (m, 1H), 1.04 (d, j = 6.6, 3H).

(*R*)-2-(2-methylpyrrolidin-1-yl)ethan-1-ol

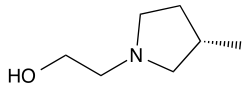

(R)-1-(2-(benzyloxy)ethyl)-3-methylpyrrolidine (1.444 g, 6.6 mmol, 1.0 equiv.) was added to a 120 mL Steel pressure vessel, methanol (60 mL) was added and the solution cooled in an ice bath for 10 minutes. 20% Pd(OH)2 on Carbon, 50 % H2O (1.422 g, 10.1 mmol, 1.5 equiv.) was added to the cooled solution and flushed with nitrogen. The vessel was sealed, evacuated and pressurized to 120 psi with hydrogen. This suspension was stirred for 1 day. A sample was taken after one day and the TLC indicated very litle reaction had taken place. The reaction mixture was transferred to a 400 mL Parr flask and the reaction shaken for 3 days at 50 psi. A TLC of the reaction after 3 days showed that the reaction had not progressed. Possible poisoning of the catalyst from the steel pressure vessel. The vessel was cleaned with a solution of Oxone and rinsed with methanol. The reaction mixture was filtered through Celite and concentrated to an oil (1.27 g recovered). The oil was added to the pressure vessel, dissolved into methanol (60 mL) and 20 % palladium hydroxide on carbon (1.27 g) was added to the reaction. The pressure vessel was purged with hydrogen (3 x) with vigorous stirring and pressurized to 140 psi. The reaction was stirred for 5 hours. TLC analysis of the reaction mixture showed very little of the product had formed. The mixture was filtered through Celite and concentrated to an oil. The oil was dissolved into methanol (60 mL) and added to a 400 mL Parr bottle. 20 % Palladium hydroxide on carbon (1.25 g) was added to the flask and the system flushed with hydrogen (3 x) and pressurized to 97 psi and left to shake for 1.5 days. TLC analysis indicated partial reduction had occurred. Hydrochloric acid (1 M, 5.5 mL) was added to mixture and the flask was flushed with hydrogen and repressurized to 97 psi. The flask was shaken for 16 hours. TLC analysis indicated the reaction was complete. Celite (3 g) was added to the Parr flask and the mixture

filtered through a pad of Celite (2 cm). The solid was washed alternating between methanol (3 x 50 mL) and water (3 x 20 mL). The filtrate was concentrated on a rotovap to dryness. The residue was taken up in methanol (10 mL) and 25 % sodium methoxide in methanol (1.17 mL, 5.4 mmol, 0.98 equiv) was added to the methanolic solution to give a white suspension. The mixture was concentrated to dryness and taken up into DCM (10 mL). The suspension was filtered through 0.45 µm PTFE frit and concentrated to a yellow oil. The oil was redissolved into DCM (10 mL), filtered (0.45 µm PTFE frit) and concentrated to furnish a yellow oil (0.5044 g, 59.2 %). This material was used without further purification.

(3*S*)-3-methyl-1-(2-(4-(4-methyl-7-((tetrahydro-2*H*-pyran-2-yl)oxy)-3-(4-((tetrahydro-2*H*-pyran-2-yl)oxy)phenyl)-2*H*-chromen-2-yl)phenoxy)ethyl)pyrrolidine

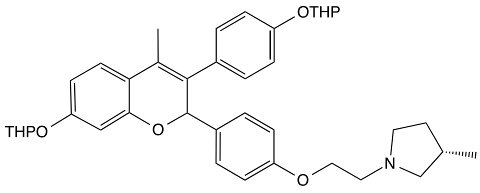

A mixture of 2-(4-iodophenyl)-4-methyl-7-((tetrahydro-2H-pyran-2-yl)oxy)-3-(4-((tetrahydro-2H-pyran-2-yl)oxy)phenyl)-2H-chromene (0.970 g, 1.6 mmol, 1.0 equiv.), (R)--2-(3-methylpyrrolidin-1-yl)ethanol (600 mg, 4.6 mmol, 3.0 equiv.), 1,10-Phenanthroline (56 mg, 0.3 mmol, 0.2 equiv.), and Cesium carbonate (1.012 g, 3.1 mmol, 2.0 equiv.) in butyronitrile (4.5 mL) was charged into a sealable tube (20 mL) and the tube was evacuated and backfilled with argon, Copper(I) iodide (296 mg, 1.6 mmol, 1.0 equiv.) was added to the mixture and evacuated and backfilled with argon three times. The reaction mixture was heated at 125°C for 40 h. HPLC showed the completion of the reaction. The reaction was cooled to room temperature and diluted with DCM and the mixture was passed through a Celite pad, which was washed with DCM, EA

and MeOH. The filtrate was collected and concentrated. The residue was purified on a silica gel column to yield the titled compound as a brown powder. The product was characterized by MS ($C_{39}H_{48}NO_6$, M+1, calc'd = 326.3, obs. 326.5) ($C_{34}H_{40}NO_5$, M+1-THP, calc'd = 342.3, obs. = 342.3).

<u>3-(4-hydroxyphenyl)-4-methyl-2-(4-(2-((*S*)-3-methylpyrrolidin-1-yl)ethoxy)phenyl)-2*H*-chromen-7-ol (OP-1046).</u>

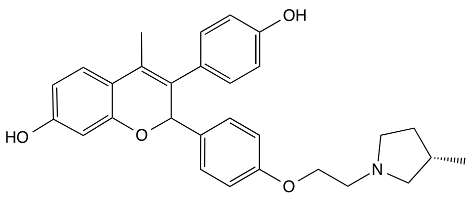

The stirred solution of (3S)-3-methyl-1-(2-(4-(4-methyl-7-((tetrahydro-2H-pyran-2-yl)oxy)-3-(4-((tetrahydro-2H-pyran-2-yl)oxy)phenyl)-2H-chromen-2-yl)phenoxy)ethyl)pyrrolidine (0.740 g, 1.2 mmol, 1.0 equiv.) in 80% acetic acid/water (50 ml) was heated to 90°C for 50 min. HPLC showed the completion of the reaction. The reaction was concentrated and the residue was neutralized with sat'd aq. $NaHCO_3$ (50 ml x 2) and extracted with EA (200 ml x 2). The organic layer was washed with brine, dried over anhy. Na_2SO_4, filtered and concentrated to give a red residue which was subjected to a silica gel purification (0 - 10% MeOH/DCM) to afford the titled compound as a red powder. HPLC: 98.8% purity (0 - 90% acetonitrile/water). LC_MS: $[M+1]^+$ = 458.2. ^1H NMR, (300 MHz, $DOCD_3$) δ 7.19 (d, j = 8.7, 2H), 7.12 (d, j = 8.1, 1H), 6.98 (d, j = 8.7, 2H), 6.77 (d, j = 8.7, 2H), 6.70 (d, j = 8.4, 2H), 6.34 (d, j = 8.1, 1H), 6.11 (d, j = 2.4 1H), 5.78 (s, 1H), 4.11 (t, j = 5.7, 2H), 3.00 (t, j = 6.6, 7.0, 1H), 2.92-2.80 (m, 2H), 2.69-2.58 (m, 1H), 2.35-2.11 (m, 2H), 2.03 (s, 3H), 1.46-1.34 (m, 1H), 1.05 (d, j = 6.6 3H).

(*S*)-2-(benzyloxy)-1-(2-methylpyrrolidin-1-yl)ethan-1-one

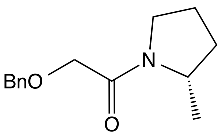

90.0% (S)-2-Methylpyrrolidine (0.441 g, 4.7 mmol, 1.0 equiv.) was added to a round bottom flask and dissolved into anhydrous DCM (20 mL). Dry Diisopropylethylamine (1.000 ml, 5.7 mmol, 1.2 equiv.) and activated 4 Å molecular sieves (~ 5 g) was added to the solution and stirred for 10 minutes. 2-(Benzyloxy)acetyl chloride (1.021 g, 5.5 mmol, 1.2 equiv.) dissolved into DCM (5 mL) was added to the reaction at room temperature dropwise via syringe over 5 minutes with a room temperature water bath for cooling. After complete addition the reaction was stirred for 3 hours. The reaction mixture became yellow over 30 minutes. TLC analysis (10 % MeOH/DCM, Crude R_f: 0.84, 0.47; SM R_f 0.26, 0.08) showed no presence of amine. The reaction was poured into a separatory funnel and the organic layer washed successively with 1 M HCl (2 x 25 mL), saturated sodium bicarbonate (25 mL) and brine (25 mL). The organic layer was dried over anhydrous $MgSO_4$, filtered and concentrated to an orange oil (1.3104 g, Y9669). The oil was loaded onto silica gel (3 g) washed through with methanol/DCM and concentrated to give a yellow oil (1.1 g, 101.1 %). This material was used in the following reaction.

(*S*)-1-(2-(benzyloxy)ethyl)-2-methylpyrrolidine

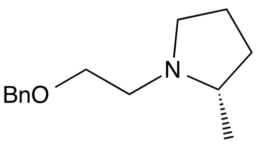

Aluminum trichloride (1.865 g, 14.0 mmol, 3.0 equiv.) was suspended into anhydrous THF (30 mL) and cooled in an ice bath. Lithium aluminum hydride (1.221 g, 32.2 mmol, 6.9 equiv.) was added in small portions to the above suspension and stirred for 10 minutes. The suspension was cooled to -78 °C for 5 minutes and a solution of (S)-2-(Benzyloxy)-1-(2-methylpyrrolidin-1-yl)ethanone (1.088 g, 4.7 mmol, 1.0 equiv.) in anhydrous THF (30 mL) was added dropwise to the cold suspension. The reaction was kept at -78 °C for 1 hour and stirred at room temperature for 2 hours. The reaction was carefully quenched with 6 N HCl solution (5 mL) and stirred for 10 minutes to give grey aqueous layer. A solution 6 N NaOH (7.5 mL) was added to the mixture to give a grey white mixture of solid in the aqueous layer. The mixture was filtered through a pad of Celite (2 cm). The solids were washed with DCM (5 x 50 mL). The filtrate was cooled in a -78 °C bath until the aqueous portion froze to ice. The organic layer was poured into a Erlenmeyer flask. The ice was melted and poured into a separatory funnel (~3 mL aqueous layer recovered) and extracted with DCM (3 x 15 mL). The organic layers were combined and dried over anhydrous sodium sulfate, filtered and concentrated to a faint yellow solid (1.0495 g). This solid was dissolved into DCM (5 mL) and loaded onto silica gel (2.5 g) and chromatographed through silica gel (25 g cartridge) with 50-100 % ethyl acetate in hexanes followed by 10-40 % methanol in dichloromethane to give a white solid (0.5891 g, 57.6 %) ^{1}H NMR, (300 MHz, $CDCl_3$) δ 77.38-7.25 (m, 5H), 4.56 (s, 3H), 3.83-3.65 (m, 2H), 3.43-3.37 (m, 1H), 3.24-3.14 (m, 1H), 2.78-2.47 (m, 3H), 2.06-1.58 (m, 4H), 1.28)d, j = 6, 3H). see: Lowe III, J.A. et al., J. Med. Chem. 2004, 47, 1575.

<u>(*S*)-2-(3-methylpyrrolidin-1-yl)ethan-1-ol</u>

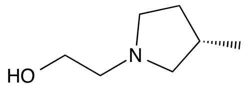

(S)-1-(2-(benzyloxy)ethyl)-2-methylpyrrolidine (0.589 g, 2.7 mmol, 1.0 equivwas added to a 400 mL Parr flask, methanol (60 mL) was added and the solution cooled in an ice bath for 10 minutes. 20% Pd$(OH)_2$ on Carbon, 50 % H2O (0.377 g, 2.7 mmol, 1.0 equiv.) was added to the cooled solution and flushed with nitrogen. Hydrochloric acid (1 M, 2.70 mL) was added to mixture. The flask was pressurized with hydrogen to 30 psi shaken for 1 minute and the hydrogen released. This was repeated twice more and pressurized to 100 psi with hydrogen. This suspension was shaken for 16 hours. A sample was taken and the TLC (10 % MeOH in DCM) indicated the reaction was complete and Celite (3 g) was added to the Parr flask and the mixture filtered through a pad of Celite (2 cm). The solid was washed alternating between methanol (2 x 25 mL) and water (2 x 25 mL). The filtrate was concentrated on a rotovap to dryness to give a white solid (0.49 g). The solid was taken up in methanol (10 mL) and 25 % sodium methoxide in methanol (0.59 mL, 2.69 mmol, 1 equiv) was added to the methanolic solution to give a white suspension. The mixture was concentrated to dryness and taken up into anhydrous DCM (10 mL). The suspension was filtered through a 0.45 µm PTFE frit and concentrated to a yellow oil. The oil was redissolved into DCM (10 mL), filtered (0.45 µm PTFE frit) and concentrated to a yellow liquid (0.2678 g, 77.2 %). ^{1}HNMR (300 MHz, CDCl3): δ3.5-3.7 (m, 2 H), 3.12-3.19 (dt, *J* = 3.3, 9.3 Hz, 1H), 2.99-3.02 (m, 1H), 2.87 (bs, 1H), 2.46 (sext, *J* = 7.0 Hz, 1H), 2.25 (dt, *J* = 3.3, 12 Hz, 1 H), 2.15 (quart, *J* = 8.7 Hz, 1H), 1.97 (m, 2H), 1,72 (m, 2H), 1.40 (m, 1H), 1.1 (d, *J* = 6.3 Hz)

(2*S*)-2-methyl-1-(2-(4-(4-methyl-7-((tetrahydro-2*H*-pyran-2-yl)oxy)-3-(4-((tetrahydro-2*H*-pyran-2-yl)oxy)phenyl)-2*H*-chromen-2-yl)phenoxy)ethyl)pyrrolidine

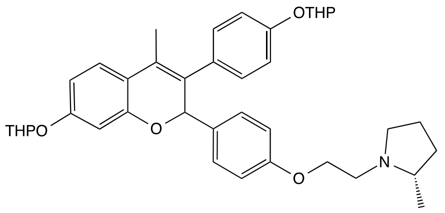

A mixture of 2-(4-iodophenyl)-4-methyl-7-((tetrahydro-2H-pyran-2-yl)oxy)-3-(4-((tetrahydro-2H-pyran-2-yl)oxy)phenyl)-2H-chromene (0.400 g, 0.6 mmol, 1.0 equiv.), -(S)-2-(2-methylpyrrolidin-1-yl)ethanol (248 mg, 1.9 mmol, 3.0 equiv.), 1,10-Phenanthroline (23 mg, 0.1 mmol, 0.2 equiv.), and Cesium carbonate (0.417 g, 1.3 mmol, 2.0 equiv.) in butyronitrile (1.9 mL) was charged into a sealable tube (10 mL) and the tube was evacuated and backfilled with argon, Copper(I) iodide (122 mg, 0.6 mmol, 1.0 equiv.) was added to the mixture and evacuated and backfilled with argon three times. The reaction mixture was heated at 125°C for 40 h. HPLC showed the completion of the reaction. The reaction was cooled to room temperature and diluted with DCM and the mixture was passed through a Celite pad which was washed with DCM multiple times. The filtrate was collected and concentrated. The residue was passed through a silica gel plug to yield the titled compound as a brown powder. LCMS confirmed structure. $C_{39}H_{48}NO_6$ (M+1) calc'd. = 626.34, obs. = 626.3.

<u>3-(4-hydroxyphenyl)-4-methyl-2-(4-(2-((*S*)-2-methylpyrrolidin-1-yl)ethoxy)phenyl)-2*H*-chromen-7-ol (OP-1047)</u>

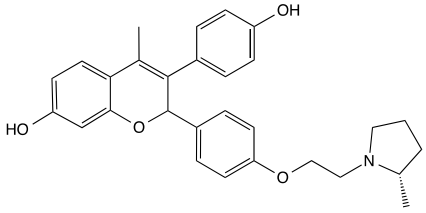

The stirred solution of (2S)-2-methyl-1-(2-(4-(4-methyl-7-((tetrahydro-2H-pyran-2-yl)oxy)-3-(4-((tetrahydro-2H-pyran-2-yl)oxy)phenyl)-2H-chromen-2-yl)phenoxy)ethyl)pyrrolidine (0.307 g,

0.5 mmol, 1.0 equiv.) in 80% acetic acid/water (50 ml) was heated to 90°C for 50 min. HPLC showed the completion of the reaction. The reaction was concentrated and the residue was neutralized with sat'd aq. $NaHCO_3$ (50 ml x 2) and extracted with EA (200 ml x 2). The organic layer was washed with brine, dried over anhy. Na_2SO_4, filtered and concentrated to give a red residue which was subjected to a silica gel purification (0 - 10% MeOH/DCM) to afford the titled compound as a red powder (168 mg, 74.8%). ^1H NMR, (300 MHz, $DOCD_3$) □ 7.19 (d, j = 8.4, 2H), 7.11 (d, j = 8.1, 1H), 6.98 (d, j = 8.7, 2H), 6.77 (d, j = 8.4, 2H), 6.69 (d, j = 8.4, 2H), 6.34 (dd, j = 2.4, 8.4, 1H), 6.11 (d, j = 2.4, 1H), 5.78 (brs, 1H), 4.10-4.03 (m, 2H), 3.37-3.17 (m, 2H), 2.62-2.48 (m, 2H), 2.37 (q, j = 8.7, 1H), 2.10-1.93 (m, 1H), 2.04 (s, 3H), 1.83-1.78 (m, 2H), 1.50-1.41 (m, 1H), 1.15 (d, j = 6.3, 3H). HPLC: 100% purity (0 - 90% acetonitrile/water). LC_MS: $C_{29}H_{32}NO_4$ $[M+1]^+$ calc'd. = 458.23, obs. = 458.3.

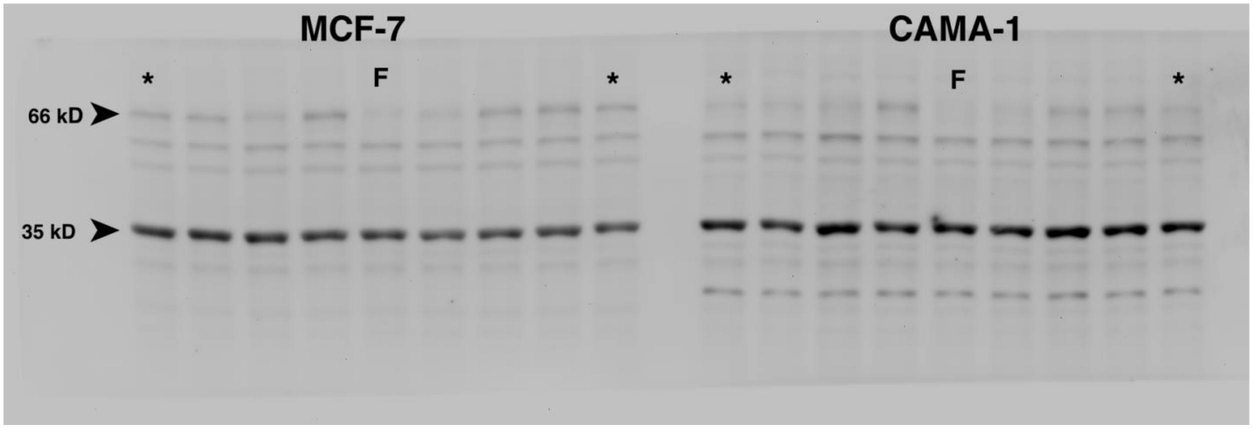

Supplementary Figure 5

Image of full original western blot shown in figure 2D. Asterisks denote additional vehicle-treated lanes that were cropped out of the final image. Sizes displayed for each band of interest, 66 kD for ER α and 35 kD for β-actin, matched those reported by the antibody manufacturer. The identity of the ER α band was confirmed by the decrease in signal mediated by treatment with fulvestrant (F).

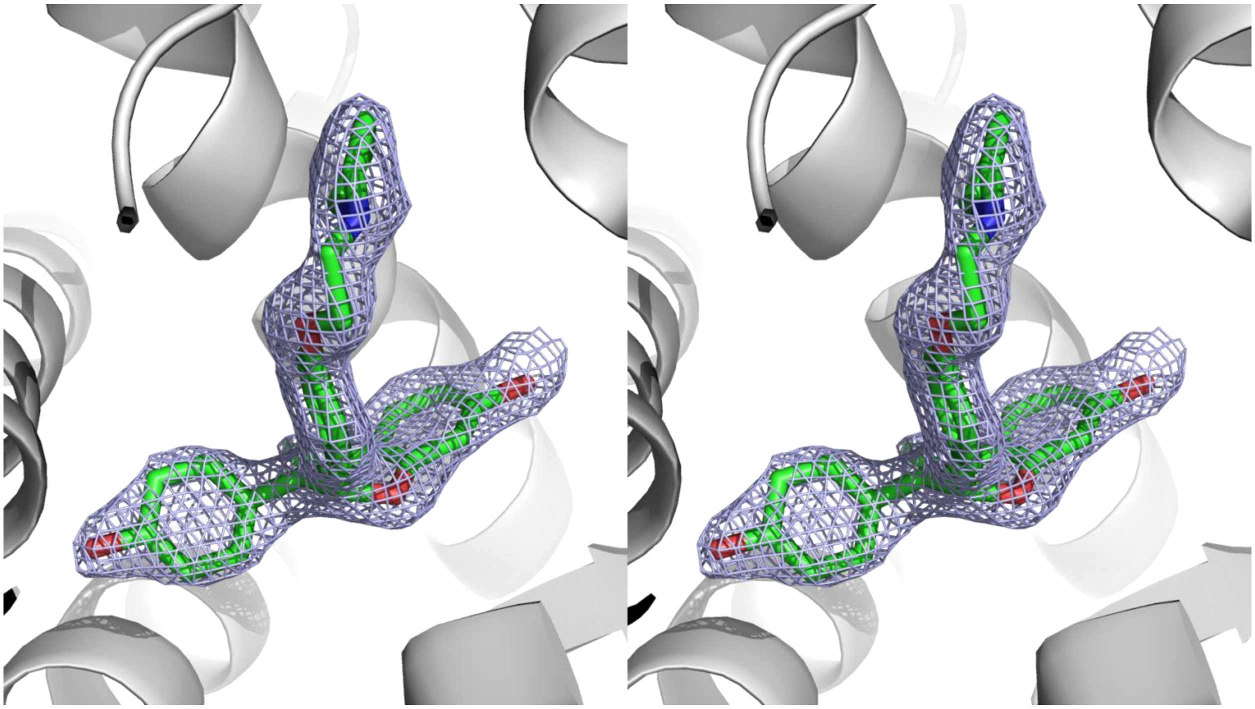

Supplementary Figure 6

Stereo-view of a simulated annealing composite omit map of OP-1156 contoured to 1.5 σ (blue mesh). OP-1156 is shown as green sticks and protein is shown as white ribbons.

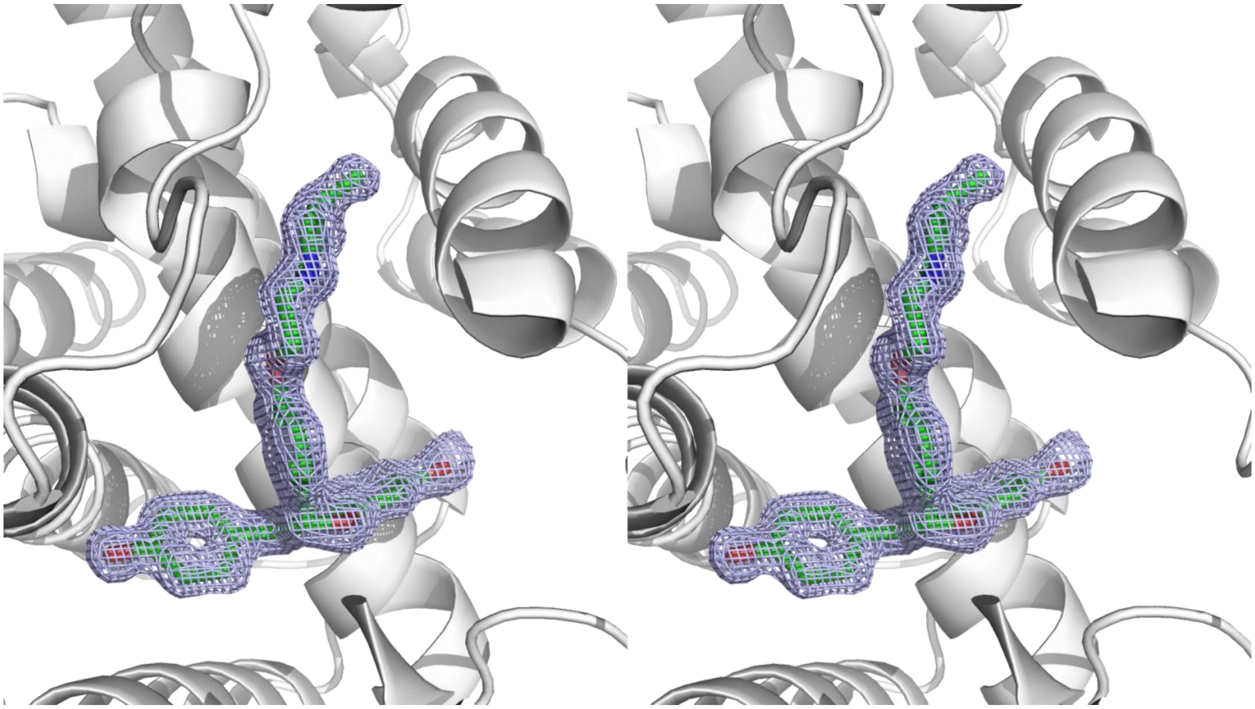

Supplementary Figure 7

Stereo-view of a simulated annealing composite omit map of OP-1154 contoured to 1.5 σ (blue mesh). OP-1154 is shown as green sticks and protein is shown as white ribbons.

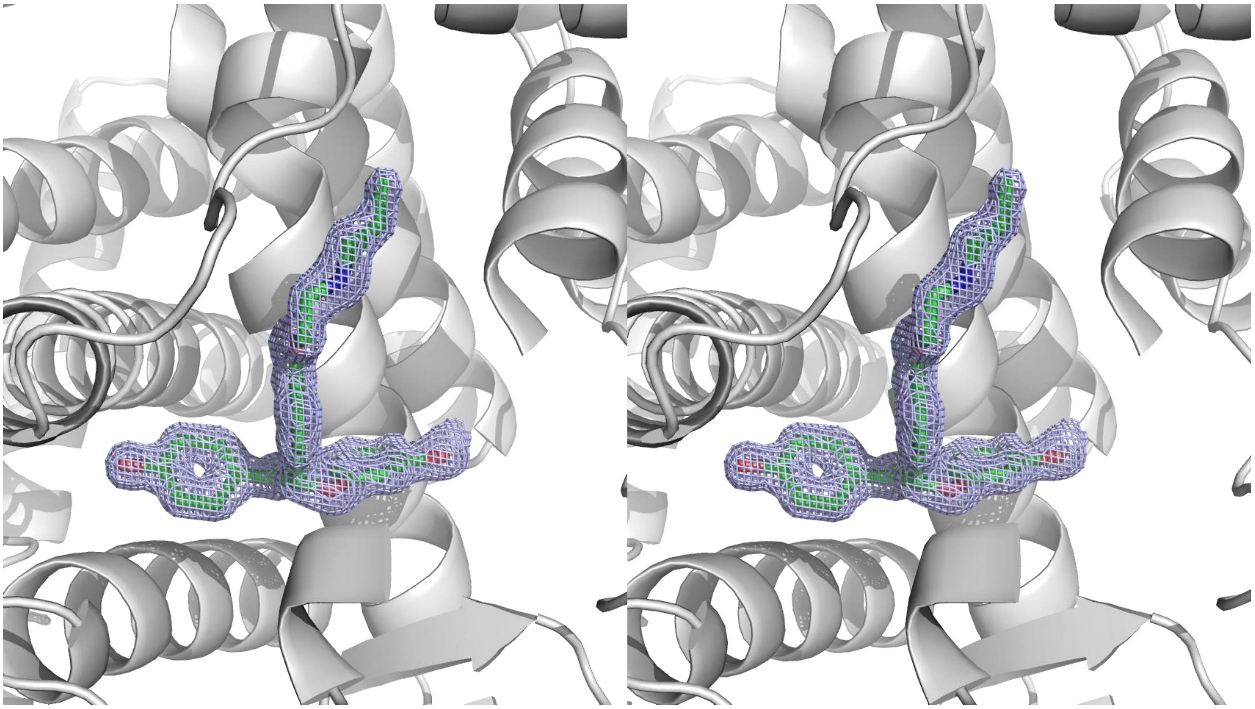

Supplementary Figure 8

Stereo-view of a simulated annealing composite omit map of OP-1074 contoured to 1.5 σ (blue mesh). OP-1074 is shown as green sticks and protein is shown as white ribbons.
